# “Drugs to avoid” to improve quality use of medicines: how is Australia faring?

**DOI:** 10.1186/s40545-021-00346-3

**Published:** 2021-07-13

**Authors:** Agnes Vitry, Barbara Mintzes

**Affiliations:** 1 Clinical and Health Sciences, University of South Australia, GPO Box 2471, Adelaide, SA 5001 Australia; 2 Charles Perkins Centre and School of Pharmacy, University of Sydney, Camperdown, Sydney Australia

**Keywords:** Medicine safety, Quality use of medicines, Harm–benefit balance

## Abstract

**Background:**

Each year, the French independent bulletin *Prescrire* publishes a list of medicines, “Drugs to avoid”, that should not be used in clinical practice as their risk-to-benefit ratio is unfavourable. This study assessed the market approval, reimbursement and use of these medicines in Australia.

**Methods:**

The approval status of the medicines included in 2019 *Prescrire* “Drugs to avoid” list was assessed by searching the Australian Register of Therapeutic Goods website. Funding status was assessed on the Pharmaceutical Benefits Scheme (PBS) website, the Australian public insurance system. Use levels were determined by examining governmental reports on prescribing rates including the Australian Statistics on Medicines (ASM) reports, drug use reports released by the Drug Utilisation Sub Committee (DUSC) and PBS statistics.

**Results:**

Of the 93 medicines included in the *Prescrire* 2019 “Drug to avoid” list included, 57 (61%) were approved in Australia in 2019 including 9 (16%) that were sold as over-the-counter medicines, 35 (38%) were listed on the PBS, 22 (24%) were registered but not listed on the PBS. Although most of these medicines were used infrequently, 16 (46%) had substantial use despite serious safety concerns. Dipeptidyl peptidase-4 (DPP-4) inhibitors were used by 22% of patients receiving a treatment for diabetes in 2016. More than 50,000 patients received an anti-dementia medicine in 2014, a 19% increase since 2009. Denosumab became the 8th medicine, in terms of total sales, funded by the Australian Government in 2017–2018.

**Conclusions:**

*Prescrire*’s assessments provide a reliable external benchmark to assess the current use of medicines in Australia. Sixteen “drugs to avoid”, judged to be more harmful than beneficial based on systematic, independent evidence reviews, are in substantial use in Australia. These results raise serious concerns about the awareness of Australian clinicians of medicine safety and efficacy. Medicines safety has become an Australian National Health Priority. Regulatory and reimbursement agencies should review the marketing and funding status of medicines which have not been shown to provide an efficacy and safety at least similar to alternative therapeutic options.

## Background

Adverse drug reactions (ADR) are associated with significant morbidity and mortality, placing a substantial burden on the healthcare systems in Australia and internationally. In the United States, adverse drug events were noted in 5.3% of all Medicare hospitalisations and increased by 90% from 2000 to 2008 [1]. In the state of New South Wales in Australia, the age-adjusted rates of ADR-related hospitalisations nearly doubled between 2001 and 2014 [2]. It has been estimated that there are 250,000 hospital admissions annually in Australia due to medication-related problems [3]. A number of interventions have been proposed to improve the safety of medicine use, including the identification and avoidance of potentially harmful medicines, or medicines with a poor harm–benefit balance, when safer alternative treatments are available. Although a number of tools have been developed to identify inappropriate medications [4] such as the Beers criteria or the STOPP/START criteria [5], most tools target inappropriate use in population subgroups such as older people or focus on specific drug classes such as anticholinergic drugs.

In this context, the approach proposed by *Prescrire* is unique as it proposes a list of “Drugs to avoid” by everyone. *Prescrire* is a French journal which has assessed the value of new medicines marketed in Europe and in France since 1981. It is exclusively financed through subscriptions and does not accept advertising or other external financial support. All *Prescrire* reviews are undertaken by trained healthcare professionals totally free from conflicts interests and are based on a comprehensive search of peer-reviewed literature and regulatory assessment reports. These reviews are then peer-reviewed by an extensive network of external reviewers [6]. The quality and thoroughness of *Prescrire*’s assessments are well recognised not only in France but internationally and their findings are used in international studies [7].

The conclusions of *Prescrire*’s assessments sometimes differ from those reached by regulatory authorities such as the European Medicines Agency due to *Prescrire’*s focus on clinical benefits and harms. Concerns have been raised that a significant proportion of medicines are introduced to the market in the absence of any proof of tangible clinical benefit for patients [8–11]. Current regulatory evidence standards allow the use of unvalidated surrogate markers for efficacy in marketing applications. Regulatory approvals may be based on biased or non-comparative clinical trials and speedier approval processes that do not provide sufficient time to assess medicines rigorously [12]. Regulatory approvals are not regularly reviewed when new evidence or better alternative treatments become available. Furthermore, there is no universally accepted standard for what constitutes a favourable harm–benefit balance, i.e. the threshold at which benefits such as prevention of cardiovascular disease or pain reduction outweigh harm from severe adverse effects. This is reflected in differences between regulators in drug safety withdrawal decisions [12]. There can also be differences in judgments about the amount of evidence needed and extent of uncertainty, and *Prescrire*’s conclusions may differ from those reached by regulators when it judges that patients’ interests are better served by a more thorough evaluation and more robust efficacy data, including in more diverse patient populations, reflecting the range of patients who will use the medicine in clinical care. *Prescrire* also values the safety of patients highly, and consistently recommends against use of medicines with no established effectiveness advantage over alternative treatments but a higher risk of harm.

Based on these reviews, each year *Prescrire* publishes a list of “Drugs to avoid” that are considered to be more harmful than beneficial. These are medicines with adverse effects that outweigh benefits, older medicines that have been superseded by medicines with a better harm–benefit balance, new medicines that are less effective or more harmful than existing alternatives, and medicines without proven efficacy but which may expose people to serious adverse effects [13]. The aim of this list is to provide a simple-to-use tool for clinicians that helps them to avoid unnecessarily hazardous prescriptions.

In this study, we have assessed how medicines included in *Prescrire*’s 2019 list of “Drugs to Avoid” are approved, publicly reimbursed, and prescribed in Australia. In Australia, use of medicines is regulated by the Therapeutics Goods Administration (TGA) that assesses their risks and benefits, based on evidence standards similar to those of other regulatory authorities such as the European Medicines Agency. As in Europe and the United States, only a minority of new medicines approved in Australia have been shown to have added therapeutic value as compared to existing treatments [14]. Medicines approved by the TGA are then evaluated for funding on the Pharmaceutical Benefits Scheme (PBS), Australia’s public insurance system, based on their efficacy, safety and cost-effectiveness compared to the existing standard of care. The PBS covers medicines that are used in the community (outpatient care) as well as in private hospitals. Public hospitals have separate dedicated funding for most medicines, with the exception of high-cost medicines, which are covered by the PBS.

## Methods

*Prescrire*’s 2019 list of “Drugs to avoid” covers medicines and indications approved in the European Union or in France, for which *Prescrire* published detailed evaluations over a 9-year period, from 2010 to 2018 [13]. This includes re-evaluations when substantial new evidence on efficacy or adverse effects became available.

Whether medicines included in 2019 *Prescrire* “Drugs to avoid” list were approved in Australia and their prescription-only or over-the-counter (OTC) status, were assessed by searching the Australian Register of Therapeutic Goods (ARTG) on the TGA website [15] for the active ingredients and the same indications and formulations. Funding status on the Pharmaceutical Benefits Scheme (PBS) was assessed by searching the PBS website [16]. For prescription-only medicines reimbursed by the PBS, use levels were based on reports of annual prescribing rates under the PBS and the Repatriation Pharmaceutical Benefits Scheme (RPBS, subsidisation of medicines for eligible veterans and dependants). We used 2015 data, as this was the latest Australian Statistics on Medicines (ASM) report available in May 2019, and includes data on subsidised and unsubsidised prescription medicine use expressed in Defined Daily Dose (DDD)s /1000 population/day [17]. Additionally, for classes of medicines (e.g. diabetes medicines, antidepressants, non-steroidal anti-inflammatory drugs), we expressed their usage as a percentage of total use for the class. Therapeutic classes were considered at the relevant level of the Anatomical Therapeutic Chemical Classification system, e.g. at the first level for all medicines used for the cardiovascular system (cardiology) or at the second level, drugs used for diabetes (diabetes). When available, we complemented these data with information included in the Drug Utilisation Sub Committee (DUSC) of the Pharmaceutical Benefits Advisory Committee (PBAC) drug use reports. These reports use a variety of measures to represent comparative drug usage over time [18]. Substantial rate of use was defined as use of around 10% or above within a specific therapeutic class, as expressed either in DDDs/ DDD)s/1000 population/day or % of patients treated.

## Results

*Prescrire* 2019 “Drug to avoid” list included 93 medicines that were assessed to be more harmful than beneficial for all indications approved in France or the European Union (nintedanib was mentioned separately for two indications, lung cancer and idiopathic pulmonary fibrosis, but is counted as a single medicine). In April 2019, 57 (61%) of these medicines were registered in Australia, 35 (38%) were listed on the PBS (Table 1), 22 (24%) were registered but not listed on the PBS (Table 2), 36 (39%) were not registered in Australia and 9 (16%) of these 57 were also sold as OTC medicines. Sixteen medicines had substantial rates of use. Of all patients receiving treatment for diabetes in 2016, 22% were using dipeptidyl peptidase-4 (DPP-4) inhibitors [19]. In 2014, 52,012 patients were dispensed an anti-dementia medicine, which *Prescrire* had recommended to avoid due to transient, small benefits and a serious potential for harm [20]. In 2015, four of the antidepressants included in *Prescrire* list, citalopram, escitalopram, venlafaxine and duloxetine accounted for 44% (42.2 DDD/1000 population/day) of use of new antidepressants in Australia [17]. Denosumab was ranked the 8th drug in Australia in PBS spending in 2017–2018, with 648,197 prescriptions, a 24% increase compared to 2016–2017 [21], with most new osteoporosis patients initiated on this drug [22]. *Prescrire* had highlighted limited efficacy evidence and serious adverse effects linked both to its effects on bone metabolism and the immune system.

**Table 1:** “Drugs to avoid” marketed in Australia and listed on the PBS

Therapeutic area	“Drugs to avoid” listed on PBS	Reasons to avoid	Rates of use (DDD/1000 population/day in 2015 unless otherwise indicated)
Cardiology	Olmesartan (alone or in combination with hydrochlorothiazide or amlodipine)	No more effective than other sartans but can cause severe sprue-like enteropathy [23, 24]	11.60
	Fenofibrate	No proven efficacy, cutaneous, haematological and renal adverse effects	7.14
	Ivabradine	Lack of benefit other than symptomatic for angina and heart failure, visual disturbances, cardiovascular disorders (including myocardial infarction), potentially severe bradycardia and other cardiac arrhythmias	0.17
	Nicorandil	Lack of benefit other than symptomatic for angina and heart failure, risk of severe AE	0.01
Diabetes	Dipeptidyl peptidase-4 inhibitors (DPP-4): alogliptin, linagliptin, saxagliptin, sitagliptin, vildagliptin	Efficacy on diabetes complications has not been demonstrated, risk of severe adverse effects: hypersensitivity reactions (Stevens–Johnson syndrome, anaphylaxis, bullous pemphigoid), infections, pancreatitis, intestinal obstruction	Alogliptin 0.03 Linagliptin 1.15 Saxagliptin 0.57 Sitagliptin 3.99 Vildagliptin 1.20 In July 2016, around 208,000 people were taking a gliptin, i.e. 22% of all patients treated for diabetes [19]. Gliptin use has doubled since 2012 and became the third most common treatment (alone or in combination) in 2016
	Pioglitazone	Risk of heart failure, bone fractures and bladder cancer	0.60
Alzheimer’s disease	Cholinesterase inhibitors (donepezil, galantamine, rivastigmine), memantine	Transient and small benefit, risk of serious adverse effects and interactions including digestive, neuropsychiatric and cardiac adverse effects with the three cholinesterase inhibitors	Donepezil 1.23 Galantamine 0.28 Rivastigmine 0.17 Memantine 0.12 In 2014, 52,012 patients were dispensed an anti-dementia drug, a 19% increase since 2009 [20]. Most were treated for over 6 months: around 70% taking acetylcholinesterase inhibitor and 64% of people on memantine
Multiple sclerosis	Alemtuzumab	Risk of infusion-related reactions, infections, autoimmune disorders	0.02
	Natalizumab	Risk of progressive multifocal leucoencephalopathy, serious hypersensitivity reactions, liver damage	0.09
	Teriflunomide	Infections, liver damage, peripheral neuropathy	0.03
Depression	Citalopram, duloxetine, escitalopram, venlafaxine	QT prolongation and torsades de pointes with citalopram and escitalopram, risk of cardiac disorders hepatitis and severe cutaneous hypersensitivity reactions such as Stevens–Johnson syndrome with duloxetine, risk of cardiac disorders (hypertension, tachycardia, arrhythmias, QT prolongation) with venlafaxine	Citalopram 6.89 Duloxetine 4.94 Escitalopram 18.30 venlafaxine 12.06 In 2015, citalopram, escitalopram, venlafaxine and duloxetine represented 44% (42.2 DDD/1000 population/day) of use of SSRI/SNRI antidepressants in Australia [17]
Respiratory	Pholcodine	Antitussive that may cause sensitisation to neuromuscular blocking agents used in general anaesthesia	NA_OTC
	Oxymetazoline, pseudoephedrine	Decongestants for oral or nasal use with risk of serious and even life-threatening cardiovascular disorders, including hypertensive crisis, stroke and arrhythmias, as well as ischaemic colitis	NA_OTC
	Inhaled mannitol	Lack of convincing evidence of efficacy in cystic fibrosis with risk of bronchospasm and haemoptysis	0.0026
	Nintedanib	No convincing evidence of efficacy in idiopathic pulmonary fibrosis, risk of serious adverse effects including: venous thromboembolism, bleeding, hypertension, gastrointestinal perforations and impaired wound healing	NA
Gastroenterology	Cimetidine	H2 antagonist with multiple drug interactions increasing the risk of dose-dependent adverse effects of combined medicines	0.02 (0.5% of the H_2_ antagonist usage)
	Domperidone	Indicated for intractable nausea and vomiting, risk of arrhythmias and sudden death	1.43
Osteoporosis	Denosumab	Limited efficacy, risk of serious adverse effects such as musculoskeletal pain, severe infections, QT interval prolongation associated with hypocalcaemia, osteonecrosis, multiple fractures after discontinuation	8.73 The 8th drug by PBS spending in 2017–2018 with 648,197 prescriptions, a 24% increase compared to 2016–2017 [21]. Around half of initial osteoporosis treatment was with denosumab in 2015 (35,260 patients); with the rest of use (47,303 patients) in previous users of at least one other osteoporosis medicine [22]
Pain	Celecoxib	Greater risk of cardiovascular adverse effects (including myocardial infarction and thrombosis) and skin reactions than other NSAIDs	5.62 (25.5% of PBS prescriptions for non-steroidal anti-inflammatory drugs)
	Diclofenac/piroxicam	Greater risk of gastrointestinal and cutaneous disorders (including toxic epidermal necrolysis) than other NSAIDs	NA_OTC*
Weight loss	Orlistat	Gastrointestinal disorders, liver damage, hyperoxaluria, and bone fractures in adolescents	0.00044
Smoking cessation	Bupropion	Neuropsychiatric disorders, potentially severe allergic reactions (including angioedema and Stevens–Johnson syndrome), addiction, and congenital heart defects in children exposed to the drug in utero	0.04871
Skin/allergy	Promethazine injection	Thrombosis, skin necrosis and gangrene following extravasation or inadvertent injection into an artery	0.0377

*DDD* defined daily dose, *NA* rate of use not available, *NA_OTC* rates of use not available as these medicines can be also sold over-the-counter

**Table 2:** “Drugs to avoid” marketed in Australia but not listed on the PBS

Therapeutic area	“Drugs to avoid” marketed but not listed on PBS	Reasons for avoiding
Cardiology	Bezafibrate	No proven efficacy in the prevention of cardiovascular events, cutaneous, haematological and renal adverse effects
	Ranolazine	Gastrointestinal and neuropsychiatric disorders, palpitations, bradycardia, hypotension, QT prolongation and peripheral oedema
Endocrinology	Conjugated oestrogens + bazedoxifene	Risks of thrombosis and hormone-dependent cancers have not been adequately evaluated
	Tibolone	Risk of cardiovascular disorders, breast cancer and ovarian cancer
Gynaecology	Ulipristal	Serious liver injury
Gastroenterology	Droperidol	Ventricular arrhythmias and sudden death
	Glyceryl trinitrate ointment	No proven efficacy for chronic anal fissure, headache that can be severe
	Prucalopride	Adverse effect profile is poorly documented, particularly with respect to cardiovascular disorders, depression and suicidal ideation and teratogenicity
Infections	Moxifloxacin	Toxic epidermal necrolysis and fulminant hepatitis and has also been linked to an increased risk of cardiac disorders
Oncology	Nintedanib	Indicated for non-small cell lung cancer serious adverse effects including venous thromboembolism, bleeding, hypertension, gastrointestinal perforations and impaired wound healing
	Panobinostat	Many, often serious and life-threatening adverse effects
	Vandetanib	Serious adverse effects (diarrhoea, pneumonia, hypertension) occur in about one-third of patients. There is also a risk of interstitial lung disease, torsades de pointes and sudden death
	Vinflunine	High risk of haematological adverse effects (including aplastic anaemia), and a risk of serious infections and cardiovascular disorders (torsades de pointes, myocardial infarction, ischaemic heart disease)
Pain	Etoricoxib	Excess of cardiovascular events (including myocardial infarction and thrombosis) and skin reactions
	Glucosamine	Allergic reactions (angioedema, acute interstitial nephritis) and hepatitis
	Parecoxib	Excess of cardiovascular events (including myocardial infarction and thrombosis) and skin reactions
Psychiatry	Agomelatine	No proven efficacy, hepatitis and pancreatitis, suicide and aggression, as well as serious skin disorders including Stevens–Johnson syndrome
	Dapoxetine	Aggressive outbursts, serotonin syndrome, and syncope
	Milnacipran	Cardiac disorders (hypertension, tachycardia, arrhythmias, QT prolongation)
Respiratory medicines	Bromhexine	Risk of anaphylactic reactions and severe, sometimes fatal cutaneous reactions such as erythema multiforme, Stevens–Johnson syndrome and toxic epidermal necrolysis
	Phenylephrine	Serious and even life-threatening cardiovascular disorders, including hypertensive crisis, stroke and arrhythmias, as well as ischaemic colitis
Weight loss	Bupropion + naltrexone	Neuropsychiatric disorders (including aggressiveness, depression and suicidal ideation), potentially severe allergic reactions (including angioedema and Stevens–Johnson syndrome), addiction, and congenital heart defects in children exposed to the drug in utero

## Discussion

The results show that 35 medicines included in the *Prescrire* 2019 “Drug to avoid” list are listed on the PBS including 16 medicines (46%) with substantial use: the five DPP-4 inhibitors for diabetes, four medicines indicated in the treatment of Alzheimer’s disease, four antidepressants, denosumab in osteoporosis, olmesartan and celecoxib.

DPP-4 inhibitors are recommended for use as second- or third-line treatment for type 2 diabetes in several international guidelines and in Australian guidelines [25]. However, DPP-4 inhibitors only provide a modest decrease in glycated haemoglobin without evidence of benefits on complications of diabetes, including cardiovascular outcomes, demonstrated in randomised clinical trials. DPP-4 inhibitors can cause severe adverse effects such as hypersensitivity reactions (Stevens–Johnson syndrome, anaphylaxis, bullous pemphigoid), infections, pancreatitis or intestinal obstruction. Despite these concerns, DPP-4 inhibitors are used widely in Australia and in other industrialised countries. In 2016, DPP-4 inhibitors were the most common second‐line drug (43% prescriptions) in England [26] and represented 20% of adjunctive treatment post-metformin initiation in the United States [27]. Several factors may explain differences in rates of uptake of new diabetes medicines between countries including variations in local clinical guidelines, economic considerations [28] and promotional campaigns by pharmaceutical companies, including industry payments to health professionals [29, 30]. Most importantly, demonstration of benefit with regard to improved glucose control, a surrogate outcome, remains the principal clinical outcome required by regulatory authorities for approval of diabetes medicines [31]. New safety signals may lead to measures to mitigate risks, such as updating the product information and/or issuing safety warnings, but they very rarely lead to market withdrawal [32].

The four drugs marketed for Alzheimer’s disease including three cholinesterase inhibitors (donepezil, galantamine, rivastigmine) and memantine have transient and limited efficacy [33]. In Australia, DUSC expressed concerns with the increasing use of these medicines (19% increase between 2009 and 2014), in particular their use for long periods of time or in combination with other anticholinergic medicines [20]. Cholinesterase inhibitors and memantine are the medications most often used in reported adverse drug reactions in people with dementia [34]. In 2016, the French regulatory authority decided to remove these medicines from the list of reimbursable products given the absence of demonstrated effectiveness, and their negative safety profile [33]. A guideline has been released in Australia to help with deprescribing these medicines in dementia [35]. It is not known yet if this guideline will have a substantial impact on prescribing.

Denosumab has limited efficacy in terms of fracture prevention in osteoporosis and has serious adverse effects such QT interval prolongation associated with hypocalcaemia and severe infections. Between 2010 and 2018, thousands of cases of immune dysfunction with denosumab have been reported globally [36]. There is a high risk of multiple vertebral fractures after denosumab discontinuation [37]. Since its first marketing in Australia to March 2021, 313 cases of hypocalcaemia have been reported with denosumab to the Therapeutics Goods Administration including 19 deaths, and also 76 cases of cellulitis, 315 cases of osteonecrosis of the jaw, and 77 cases of atypical femoral fractures [38]. DUSC expressed concerns about frequent use among residential aged care patients who may be at higher risk of adverse effects [22]. Despite these concerns, use of denosumab continued to increase in 2017–2018, largely replacing the bisphosphonates as a first-line osteoporosis treatment [18, 22].

Duloxetine, citalopram, escitalopram and venlafaxine are antidepressants which represented almost half of antidepressant use in Australia in 2015 [17]. They are all among the medicines recommended as initial treatment options for major depression by Australian Therapeutic guidelines [39]. Although these guidelines state that they may cause a wide range of adverse effects, they do not mention rare life-threatening adverse such as the QT interval prolongation and Torsades de Pointes, which can occur even at recommended doses of citalopram and escitalopram [40]. Duloxetine can cause hepatitis and severe cutaneous hypersensitivity reactions. Venlafaxine overdoses are associated with a high risk of cardiac arrest. Frequent use of antidepressants in Australia, which had the third highest consumption of OECD countries in 2017 [41], may be explained by limited access to psychological counselling, overestimation of efficacy [42] and inadequate knowledge of risks [43]. When an antidepressant is needed, safer alternatives exist to those included in the “Drugs to Avoid” list, with similar efficacy levels, including other Selective Serotonin Reuptake Inhibitors (SSRIs) such as fluoxetine, fluvoxamine, or sertraline.

Nine of the medicines to avoid (15.8%) were sold as OTC products in Australia. Although sales data were unavailable, products such as bromhexine, oxymetazoline, pseudoephedrine, pholcodine are ingredients in cough and cold products, and glucosamine is used in arthritis. These are common conditions, suggesting frequent use.

Thirty-six (39%) of these medicines were not registered in Australia. Except in a few cases, it was not possible to determine if regulatory approval for these medicines had ever been sought in Australia. Tolcapone, a medicine indicated for Parkinson’s disease, was withdrawn two months after its regulatory approval from European countries and Australia because of the risk of severe hepatitis [44]. It was later re-introduced in European countries but not in Australia. Quinine is not marketed for nocturnal cramps in Australia and the TGA issued a safety warning for this off-label use in 2011 [45].

A study examined the marketing status of the 2017 *Prescrire*’ list of “Drugs to avoid” in Canada [46]. Most (61%) were also available in Canada, a minority of which had been assessed for therapeutic benefit in Canada (*n* = 36) as a background to pricing or reimbursement. Only 2/36 (5.5%) had been found to provide a substantial improvement.

The assessment of the therapeutic value of medicines relies on the estimation of the balance of risks and benefits. *Prescrire*’s analysis considers that there is no justification for exposing patients to medicines that may cause potentially serious or life-threatening adverse effects if safer treatment alternatives exist or in the absence of a well-demonstrated benefit. Based on their evidence review, *Prescrire* judges them to be “more dangerous than beneficial”, and believes that there is no valid reason for them to continue to be marketed. The relative value of medicines can vary from one country to another and there are differences of opinion for what constitutes an acceptable harm-to-benefit balance. Furthermore, the safety of medicines is very difficult to evaluate as it involves comparing adverse effects that can be very different qualitatively, in terms of their likelihood of occurring, and are based on different sources and levels of evidence. Nevertheless, the comparison of Australian prescribing practices versus *Prescrire*’s assessments is worth doing, as is highlights real safety concerns with Australian current prescribing practices.

National regulatory authorities vary in their decisions on market approval in regard to what represent an acceptable harm–benefit balance. One-third of the drugs in *Prescrire*’s “Drugs to Avoid” list are not currently marketed in Australia. Patterns of withdrawal of medicines because of safety reasons are inconsistent across countries [47]. There is also a low level of concordance (10.3%) between regulators in the decision to warn clinicians and the public about risks of approved prescription medicines [48]. Regulators do not publicly comment on each other’s decisions, and the reasons behind these differences are unclear. Calls have been made to raise the bar for market approval of new medicines [49], by requiring comparative evidence on meaningful clinical outcomes rather than on surrogate endpoints, in particular for medicines for prevention or chronic use, including for type 2 diabetes [50] and osteoporosis [51]. Causality of rare adverse reactions is always difficult to establish, but there is no justification for exposing patients to potential risks if safer and equally effective alternatives exist. Although DUSC has raised concerns on extensive use of several of these medicines (i.e. denosumab and drugs for Alzheimer’s disease), these concerns have not led to shifts in reimbursement or regulatory status. In a recent study, the TGA was found to be the least active in releasing medicines safety advisories compared to its counterparts in the United States, United Kingdom and Canada (no warning in 70.4% of 619 drug-risk issues identified over a 10-year period compared 41.0%, 49.9% and 52.4% in the US, Canada and the UK, respectively) [48]. Thus the extent to which Australian clinicians are warned of emergent safety concerns is an open question.

### Limitations

We have used the 2019 “Drugs to Avoid” list as we have checked the registration and funding status of the medicines in Australia in 2019. The usage data, mostly from 2015, may not reflect the safety concerns back in 2015. However, of the 35 medicines listed on the PBS, 27 (77%) were already included in the 2015 “Drugs to Avoid” list. Of the eight medicines not listed in 2015, five had a very low usage in 2015 (pioglitazone (which had been withdrawn from the French market), alemtuzumab, inhaled mannitol, nintedanib), and three were added in the 2016 list (citalopram, escitalopram and diclofenac). Safety alerts were issued for diclofenac in 2013 and additional warnings were listed in the Australian Product Information in October 2014, as requested by the TGA. The TGA also required dosing recommendations for citalopram in November 2011. Differences between the 2015 and the 2019 “Drugs to Avoid” *Prescrire*’ lists are unlikely to change the conclusions of the study.

## Conclusions

Our study showed that 16 medicines with a risk of severe adverse effects are in substantial use in Australia despite the availability of alternative treatments which are at least as effective but have a safer profile than these “drugs to avoid”. This situation is inconsistent with one of the central objectives of Australia’s National Medicines Policy, Quality Use of Medicines [52]. Quality Use of Medicines and Medicines Safety was made the 10th National Health Priority Area in Australia in 2019 [53]. Although a number of initiatives are being implemented in particular in the elderly population living in residential aged care (98% of residential aged care residents have at least one medication-related problem) [53], regulatory and funding agencies should review the evidence on benefits versus harm of marketed medicines, and take concrete steps to protect patients from medicines found to be more harmful than beneficial.

## Data Availability

All data generated or analysed during this study are included in this published article.
